# Morphologically and Functionally Distinct Lipid Droplet Subpopulations

**DOI:** 10.1038/srep29539

**Published:** 2016-07-08

**Authors:** Shuyan Zhang, Yang Wang, Liujuan Cui, Yaqin Deng, Shimeng Xu, Jinhai Yu, Simon Cichello, Ginette Serrero, Yunshu Ying, Pingsheng Liu

**Affiliations:** 1 National Laboratory of Biomacromolecules, Institute of Biophysics, Chinese Academy of Sciences, Beijing 100101, China; 2 School of Life Sciences, University of Science and Technology of China, Hefei, Anhui 230026, China; 3 University of Chinese Academy of Sciences, Beijing 100049, China; 4 School of Life Sciences, La Trobe University, Melbourne Victoria 3086, Australia; 5 A&G Pharmaceutical, Inc., Columbia, Maryland 21045, USA; 6 University of Texas Southwestern Medical Center, Dallas 75390, USA

## Abstract

Lipid droplet (LD), a multi-functional organelle, is often found to associate with other cellular membranous structures and vary in size in a given cell, which may be related to their functional diversity. Here we established a method to separate LD subpopulations from isolated CHO K2 LDs into three different size categories. The subpopulation with smallest LDs was nearly free of ER and other membranous structures while those with larger LDs contained intact ER. These distinct subpopulations of LDs differed in their protein composition and ability to recruit proteins. This method was also applicable to LDs obtained from other sources, such as Huh7 cells, mouse liver and brown adipose tissue, *et al*. We developed an *in vitro* assay requiring only isolated LDs, Coenzyme A, and ATP to drive lipid synthesis. The LD subpopulation nearly depleted of ER was able to incorporate fatty acids into triacylglycerol and phospholipids. Together, our data demonstrate that LDs in a given cell are heterogeneous in size and function, and suggest that LDs are one of cellular lipid synthetic organelles.

The lipid droplet (LD) was originally thought to be only a passive cellular storage site for excess lipids. However, in recent years, proteomic, ultra-structural and functional studies have shown that LDs are multi-functional organelles with important roles in multiple biological processes^1^,^2^. Emerging evidence also links aberrant storage of neutral lipids in LDs to human metabolic syndrome^3^. Recently, important advances have been made in LD biology but uncertainties remain, partly due to heterogeneity of LDs in size and function as well as interactions between LDs and other cellular organelles.

LDs have been shown to interact with other organelles. For example, during cholesterol import into adrenocortical cells, LDs have been shown to form complexes with lysosomes^4^. Using electron microscopy (EM), fluorescence microscopy and a reconstitution assay, our group found that LDs physically contact mitochondria and peroxisomes in skeletal muscle tissue and in cell culture^5^. Furthermore, using bimolecular fluorescence complementation in yeast, we detected 116 pairs of inter-organelle protein contact between LDs and these two organelles in yeast^6^. Direct interaction between endosomes and LDs was detected *in vitro* previously^7^. These findings suggest the involvement of LDs in intracellular lipid traffic^8^. The endoplasmic reticulum (ER) and LDs have a particularly close relationship^9^,^10^. LDs are thought to bud from the ER and electron microscopic analysis has indicated that the ER is always in close proximity to LDs^11^. Using integral membrane proteins that mark the monolayer of LDs, LDs were found to directly form from and return to ER in a cyclic manner^12^. LDs have also been described as existing in two groups, ER-associated LD (ER-LD) and cytosolic LD (C-LD)^13^. Hayashi *et al*. separated ER-LD and C-LD by sucrose gradient fractionation to study the translocation of the σ−1 receptor^14^. In that study LDs were segregated using caveolin-2 and adipocyte differentiation related protein (ADRP) to follow ER-LDs and C-LDs, respectively. Dr. Fujimoto’s group proposed that the ER membrane apposed to LD be called LD-associated membrane^10^.

The ER has long been thought to be the primary site of lipid synthesis^15^. However, recent evidence indicates that LDs have an important role in lipid homeostasis. Numerous enzymes involved in lipid metabolism have been localized on isolated LDs and on LDs in intact cells^16^,^17^,^18^,^19^,^20^,^21^. Furthermore, many studies have shown that LDs are sites for cellular lipid production^1^,^22^,^23^,^24^,^25^. Several *in vitro* studies have found lipid synthetic enzymatic activity and lipid synthesis on LDs^26^,^27^,^28^,^29^,^30^.

However, the interpretation of all of these findings is complicated by the presence of membranes from ER and other organelles in LD preparations generated by standard methods. For example, EM analysis of isolated LDs have shown adherent membranous structures^26^ and most preparations of LDs described to date, all based on the low density of LDs, have contained a significant number of ER proteins^21^,^31^,^32^,^33^,^34^,^35^,^36^,^37^,^38^.

The study of LDs is further complicated by their heterogeneity in size, a distinction that may be associated with discrete functions. Interestingly, only large LDs are found to be in contact with the ER^10^,^39^. Walther *et al*. even found large LDs in a cell could grow by addition of triacylglycerol (TAG), synthesized locally by TAG synthesis enzymes moving along the bridge from ER to LD^40^,^41^. Small LDs did not change in size due to the unavailability of these enzymes. An imbalance between large and small LDs may be a central event in the development of metabolic syndrome^42^. LDs are also proposed to be sites for protein sequestration. In this process, LDs provide a binding surface for proteins and different sized LDs afford distinct sequestration capacities and properties^43^. Biochemically dissecting the functions specific to LDs from those involving inter-organelle interaction requires a purification process that can remove all associated membranes and segregate LDs by size class. Therefore, it is clear that more advanced methods of LD purification are required to address the functions of LDs using biochemical and morphological means.

In this study, we describe a method based on differential centrifugation with which we have successfully fractionated LDs into large, medium and small subpopulations. Interestingly, the large LDs from CHO K2 cells contained more ER proteins while the small LDs were nearly ER-depleted. The purification method was applicable to LDs from Huh7 cells, mouse liver and brown adipose tissue (BAT). A simple method was developed to assay the LD-mediated lipid synthesis *in vitro* using these purified populations. The results showed that the small LDs nearly depleted of ER were capable of synthesizing lipids.

## Results

### Intact ER interacts with lipid droplets

**Figure 1:**
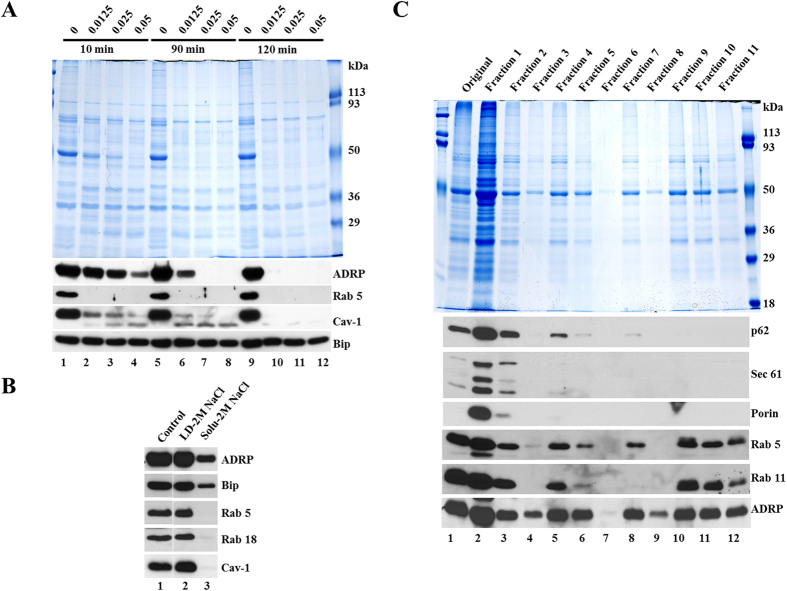
Interaction between lipid droplets and intact ER and subpopulations of lipid droplets from CHO K2 cells. (**A**) Association of intact ER to LDs. Total LDs were isolated from CHO K2 cells and distributed into 12 aliquots. The aliquots were treated with trypsin at 37 °C at the concentration (%, w/v) and times indicated. After incubation, LDs were re-isolated, and proteins were extracted with chloroform:acetone (300 μl:700 μl) and analyzed by Colloidal Blue staining and Western blotting with the indicated antibodies. (**B**) Treatment of LDs with high concentration salt. Isolated LDs were incubated with or without 2 M NaCl in Buffer B at 4 °C for 30 min. After incubation the reaction was centrifuged at 20,000 *g* for 3 min. LDs layer (top) and the solution underneath (Solu) were collected respectively. LD proteins were extracted with acetone and the solution was mixed with 5 × SDS sample buffer (to final concentration of 2 × SDS sample buffer). The proteins in LDs and solution were then analyzed by Western blotting with the indicated antibodies. (**C**) ER and mitochondrial proteins in LD subpopulations. The total LD fraction was isolated from CHO K2 cells, mixed with 2.5 M sucrose to final concentration of 1.25 M, overlaid with a discontinuous sucrose density gradient, and the gradient was centrifuged at 500 *g* for 20 min. Fractions from the top of the gradient, 1 ml each, were collected, and the proteins from those fractions were precipitated using trichloroacetic acid (final concentration of 7.2%). The precipitated proteins were separated by SDS-PAGE, and stained with Colloidal Blue or blotted with the indicated antibodies.

In previous morphological studies LDs were found to be in contact with other membrane-bound organelles, especially the ER. In agreement with these observations, biochemical studies of isolated LDs have found that ER proteins, including luminal proteins like Bip, were co-purified with LD marker proteins. To examine if LD-associated Bip was in a sealed membranous compartment, we carried out a protease protection assay to determine whether the LD-associated Bip could be digested. Isolated total LDs were treated with the indicated concentrations of trypsin, re-isolated following treatment, and the proteins were analyzed by Western blotting with the indicated antibodies. Total proteins resistant to digestion were monitored by Colloidal Blue staining (Fig. 1A). Upon trypsin treatment the LD proteins, such as ADRP, Rab 5, and caveolin-1 (Cav-1), were partially or completely digested (Fig. 1A). Rab 5 was more sensitive to trypsin digestion than Cav-1 and ADRP. In contrast, the LD-associated Bip was fully protected from digestion, suggesting that it was contained in an intact ER membrane structure that was stably associated with LDs. Similar results were obtained by using mouse liver LDs (Supplemental Fig. 1).

We next investigated whether the association between LDs and ER is based on ionic interaction. Isolated total LDs were incubated with 2 M NaCl and then re-isolated by centrifugation and washing. Since ADRP has been observed to associate with LDs via hydrophobic interactions^44^ and to bind phospholipids with high affinity^45^, it was used as a negative control. Indeed, ADRP could not be washed off by the treatment, only small amount of it detected in reaction solution (Fig. 1B). Similar to ADRP, Bip was also removed only slightly, demonstrating that portions of ER membrane tightly associate with LDs and are not easily dissociated using ionic strength. The treatment had no effect on LD-associated Rab 5, Rab 18, or Cav-1 either (Fig. 1B).

### CHO K2 cells contain subpopulations of lipid droplets

To explore potential heterogeneity in the LD population and to determine if there was a LD subpopulation not associated with ER membranes, total LDs purified by our standard method^46^ were further fractionated into 11 fractions by density gradient centrifugation as described in the Materials and Methods. Proteins in these fractions were extracted and analyzed by Western blotting and Colloidal Blue staining (Fig. 1C). LDs were distributed throughout the gradient as marked by ADRP (Fig. 1C). Marker proteins for mitochondria (Porin) and ER (Sec 61 and p62) were abundant in fractions 1–2. The remaining fractions were depleted in these marker proteins despite the presence of equivalent quantities of ADRP. No Porin, Sec 61, or p62 was detected in fractions 8–11. Interestingly, one fraction was completely depleted of Rab 11 (Fig. 1C, lane 8) and the another was negative for Rab 5 (Fig. 1C, lane 9). The results suggest the presence of distinct subpopulations of LDs including a pool of LDs almost free of ER and mitochondria.

### Separation of lipid droplet subpopulations by size

**Figure 2:**
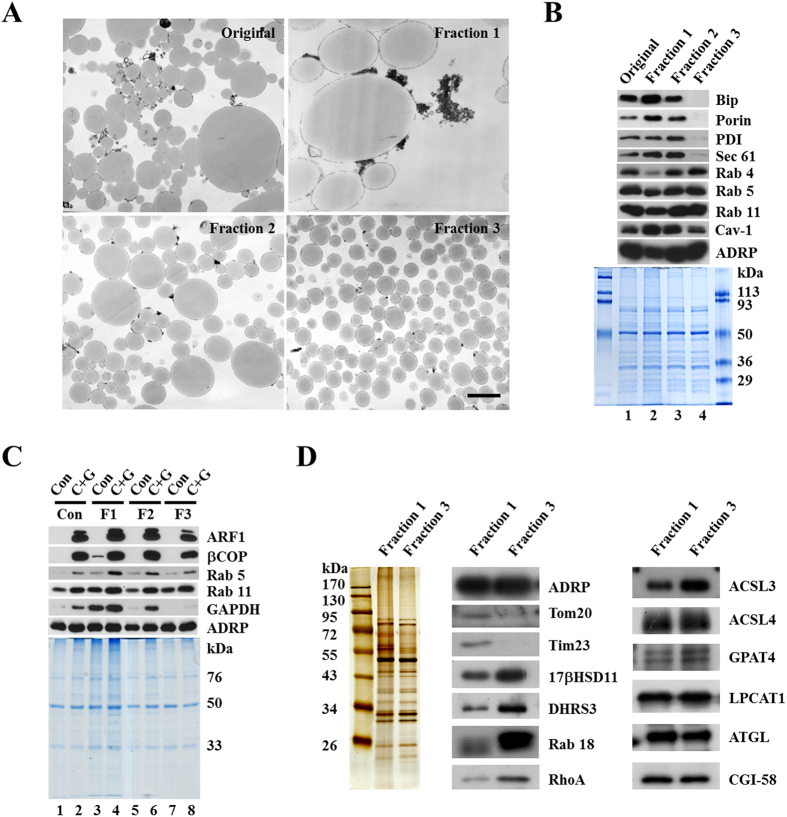
Subpopulation of lipid droplets with different sizes and protein compositions. Total LDs and cytosol were isolated from CHO K2 cells. The total LDs were further fractionated by size using differential centrifugation (Materials and Methods). LD fractions 1, 2 and 3 were collected. (**A**) LD size of the subpopulations. LD fractions were processed for fixation, dehydration and infiltration, and finally embedded in Embed 812. Then the ultra-thin sections of the three fractions were prepared and analyzed by EM. Bar = 1 μm. (**B**) ER and mitochondrial markers in the subpopulations. Proteins of these fractions were extracted with chloroform:acetone (300 μl:700 μl), separated using SDS-PAGE, analyzed by Western blotting (equal amount of protein per lane) with the indicated antibodies, and stained by Colloidal Blue. (**C**) Protein recruitment to the subpopulation. These three fractions were incubated with Buffer B (Con) or cytosol plus 1 mM GTPγs (C + G) for 1 h at 37 °C. LDs were then re-isolated and the proteins were extracted with acetone and analyzed by Western blotting with the indicated antibodies. (**D**) Protein composition of the subpopulations. The proteins of the fraction 1 and 3 were extracted with chloroform:acetone (300 μl:700 μl) and analyzed by silver staining and Western blotting (equal amount of protein per lane) with the indicated antibodies.

The existence of LD fractions lacking detectable ER and mitochondrial marker proteins raised the possibility of obtaining a subpopulation of LDs that do not associate with the ER or mitochondria. Furthermore, the finding that these distinct groups of LDs could be separated by centrifugation indicates that these LD fractions have different physical properties, either related to size or density. Therefore, we developed a multi-step method to separate these populations by size. In this method we isolated total LDs and then separated the total LDs into three distinct subpopulations by differential centrifugation, which we designate as Fractions 1 through 3, respectively (Fig. 2). As we expected, the total LDs (Original) were successfully separated into three subpopulations by size (Fig. 2A). Fraction 1 contained primarily large LDs (a few microns across) with some other adherent membrane structures, while Fraction 3 contained small LDs (less than a micron in diameter) and was almost free of other membrane structures (Fig. 2A).

### Small lipid droplets were not associated with ER or mitochondria in CHO K2 cells

The three fractions had similar total protein profiles except for Fraction 3 which had fewer high molecular weight proteins. By Western blotting, Fraction 3 had no detectable Bip, Sec 61, or PDI (ER marker proteins) and no detectable Porin (mitochondrial marker protein), while Fraction 1 was enriched in Bip and Porin (Fig. 2B). The biochemical distribution of ER and mitochondrial proteins was in agreement with the morphological findings (Fig. 2A). Fraction 3 also had less Cav-1 and Fraction 1 had substantially less Rab 4 (Fig. 2B) than the other fractions.

Using the method, we successfully obtained three subpopulations of LDs with different sizes. Importantly, the small LDs we separated from CHO K2 cells did not contain detectable ER and mitochondrial marker proteins, suggesting that they were not associated with these cellular organelles. To determine if there were functional differences among these fractions, we took advantage of *in vitro* assay that we established previously to examine the cytosolic protein recruitment ability of these subpopulations^36^. The LDs in the three fractions were incubated with cytosol in the presence or absence of GTPγs, re-isolated and washed, and analyzed for specific proteins. The LDs in all of the fractions could recruit Rab proteins, ARF1 and βCOP (Fig. 2C). However, those in Fraction 3 were not able to recruit GAPDH like the other fractions (Fig. 2C).

### Analysis of protein compositions in different lipid droplet subpopulations isolated from CHO K2 Cells

Next, we carried out a proteomic study of the three LD subpopulations to determine how they may differ in protein composition. As shown in Supplemental Fig. 2, 141 proteins were identified by MS in the three LD fractions. Approximately 45% of the proteins (63 proteins) appeared in all three fractions while 20 proteins were only found in Fraction 1, 15 only in Fraction 2, and 11 only in Fraction 3. We also compared the relative abundance of certain proteins in the fractions by analyzing the number of identified peptides in each mass spectra (Supplemental Table 1 and Supplemental Fig. 3). As shown in Supplemental Table 1, the three fractions have the same number of peptide fragments of ADRP, which is consistent with Western blotting result (Fig. 2B). This also allows ADRP to be used as an internal control. For both ER and mitochondrial resident proteins, a significant decrease could be observed from Fractions 1 to 3 (Supplemental Fig. 3A,B). The MS result also showed that membrane trafficking proteins and lipid metabolic enzymes are rich in all of the three fractions (Supplemental Fig. 3C,D). Most of the enzymes in the Kennedy pathway for the synthesis of triacylglycerol and phospholipids appear in the MS data.

We performed more extensive analysis of the protein compositions of Fraction 1 and 3 by Western blotting (Fig. 2D). With ADRP as a control, there was an obvious decrease of mitochondrial Tom20 and Tim23 in Fraction 3, further indicating a reduction in associated mitochondria. Estradiol 17-beta-dehydrogenase 11 (17βHSD11) and DHRS3 [dehydrogenase/reductase (SDR family) member 3], both belonging to short-chain dehydrogenases/reductases (SDR) family, were enriched in Fraction 3. The small G-proteins, Rab 18 and transforming protein RhoA, were much more abundant in Fraction 3 than in Fraction 1. Proteins involved in lipid metabolism, including glycerol-3-phosphate acyltransferase 4 (GPAT4), lysophosphatidylcholine acyltransferase 1 (LPCAT1), adipose triglyceride lipase (ATGL), and comparative gene identification-58 (CGI-58), were found to be on both of the LD populations with similar abundance. Two long-chain acyl-CoA synthetases, ACSL3 and ACSL4, showed different preferences to the two LD populations.

Together with the silver staining results shown in Fig. 2D, these data demonstrate that our method is effective at obtaining distinct LD subpopulations with different sizes, compositions, functional characteristics, and degree of associated organelles.

### Application of the method to other cells and tissues

**Figure 3:**
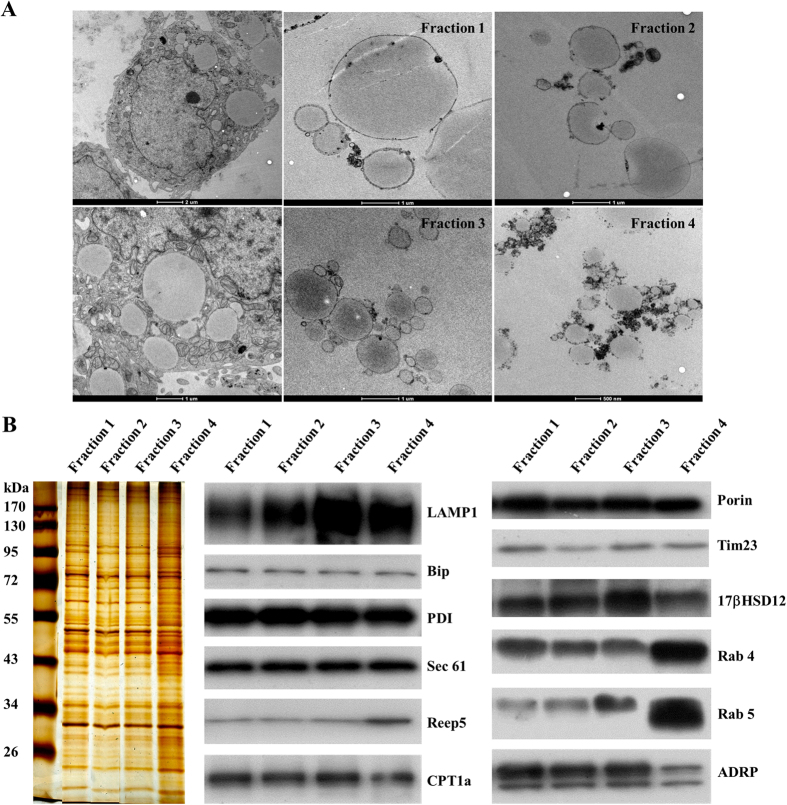
Separation of lipid droplet subpopulations from Huh7 cells by size. LDs from Huh7 cells were fractionated by size using differential centrifugation (Materials and Methods). Four LD fractions were collected. (**A**) EM to determine LD size. Huh7 cells and the four LD fractions were fixed, dehydrated and infiltrated and finally embedded for ultra-thin sectioning. The 70 nm ultra-thin sections were prepared, and the ultra-structures were analyzed by EM. (**B**) Protein composition of the subpopulations. Proteins of the four LD fractions were extracted with chloroform:acetone (300 μl:700 μl) and analyzed by silver staining and Western blotting (equal amount of protein per lane) with the indicated antibodies.

We applied this method to Huh7 cells to determine if it could be used to fractionate LDs from other sources. To avoid disruption of huge LDs present in Huh7 cells, we did not isolate total LDs by centrifugation at 182,000 *g* first. In stead, different centrifugation speeds were applied first to get all kinds of LDs and those LDs, except for Fraction 4, were combined together as a LD pool for further subpopulation. Similar to total LDs from CHO K2 cells, the LD pool were further separated into three subpopulations by differential centrifugation, designated as Fractions 1 through 3 (Fig. 3A). Using the method, the LDs in Huh7 were successfully separated into four subpopulations by size (Fig. 3A). LDs in Fraction 1 were more than 3 microns in diameter; most of LDs in Fraction 3 were less than 1 micron. Fraction 4 contained many small LDs. The pictures of the ultra structures of Huh7 cells and those isolated LDs suggested the preparation of the LD subpopulations reflected the real and diverse state of LDs in a cell. Interestingly, there was always debris surrounding those small LDs in Fraction 4. Given a similar amount of ADRP among the four fractions, ER proteins would increase dramatically in Fraction 4 (Fig. 3B). LAMP1, a lysosomal marker, showed different distributions among those fractions, suggesting the distinct interactions with lysosomes. 17βHSD12 were enriched in Fraction 3 while Rab 4 and 5 were mainly in Fraction 4. Therefore, LDs from Huh7 cells were successfully separated by size and they exhibited different degree of associated organelles.

**Figure 4:**
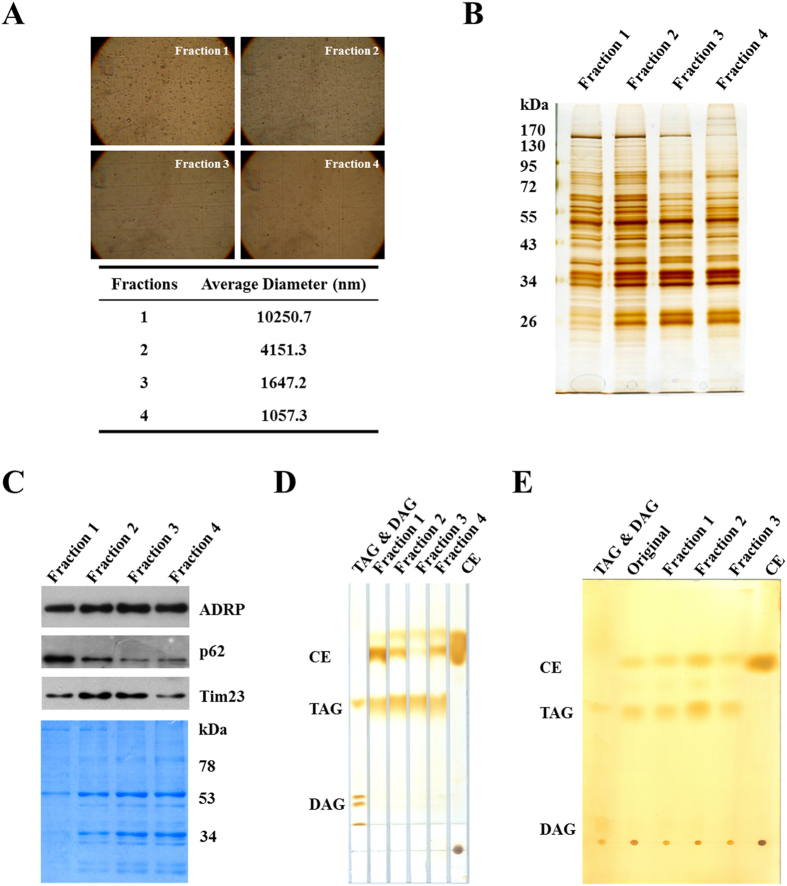
Separation and neutral lipid analysis of lipid droplet subpopulations from mouse liver. LDs from mouse liver were fractionated by size using differential centrifugation (Materials and Methods). Four LD fractions were collected. (**A**) LD size of the subpopulations. LD fractions were viewed under light microscope (upper panel). The LDs were incubated with 1% Triton for 30 min to reduce aggregation. After removing Triton, the LDs were viewed and the images were acquired at 400-fold magnification. The LD size was measured by a Delsa Nano C particle analyzer. (**B**) Protein profile of the subpopulations. The proteins of LD subpopulations were extracted and analyzed by silver staining. (**C**) ER and mitochondrial markers in the subpopulations. Proteins from the subpopulations were analyzed by Western blotting with indicated antibodies (equal amount of protein per lane). (**D**,**E**) Neutral lipid analysis. Lipids of the subpopulations from mouse liver (panel D) and CHO K2 cells (panel E) were separated by TLC with a solvent of n-hexane-diethyl ether-acetic acid (80:20:1, v/v/v). TLC plate was then stained in iodine vapor after being dried at room temperature.

We further adapted the method to mammalian tissues. Similarly, to avoid disruption of the huge LDs present in those tissues, the differential centrifugation method was applied directly to debris-clarified homogenate rather than isolated total LDs. In addition, the centrifugation speeds and the density gradient required adjustment for successful fractionation. The detailed protocols are described in the Method section. The results largely recapitulate those obtained with the cell culture derived LDs (Fig. 4). Using the method, we could fractionate LDs from mouse liver into different sizes with distinct protein profiles (Fig. 4A,B). Diameters of LDs in the four fractions were analyzed by a Delsa Nano C particle analyzer (Fig. 4A). Fraction 1 contained LDs around 10 μm, while LDs in Fraction 4 were around 1 μm. The smaller LD population had fewer proteins from other cellular organelles (Fig. 4C). Since both TAG and cholesterol ester (CE) are abundant in liver and the results suggest the presence of distinct LD pools, we analyzed the lipid profiles of the isolated LD sub-populations. Indeed, the LD fractions had different lipid profiles. Interestingly, some Fractions contained LDs lacking CE while others were enriched in this neutral lipid class (Fig. 4D). In contrast, the LDs from CHO K2 cells had similar neutral lipid compositions among the isolated Fractions (Fig. 4E). These data suggest that the heterogeneity among these LD fractions based on this method is probably due to the size and not due to the lipid composition.

**Figure 5:**
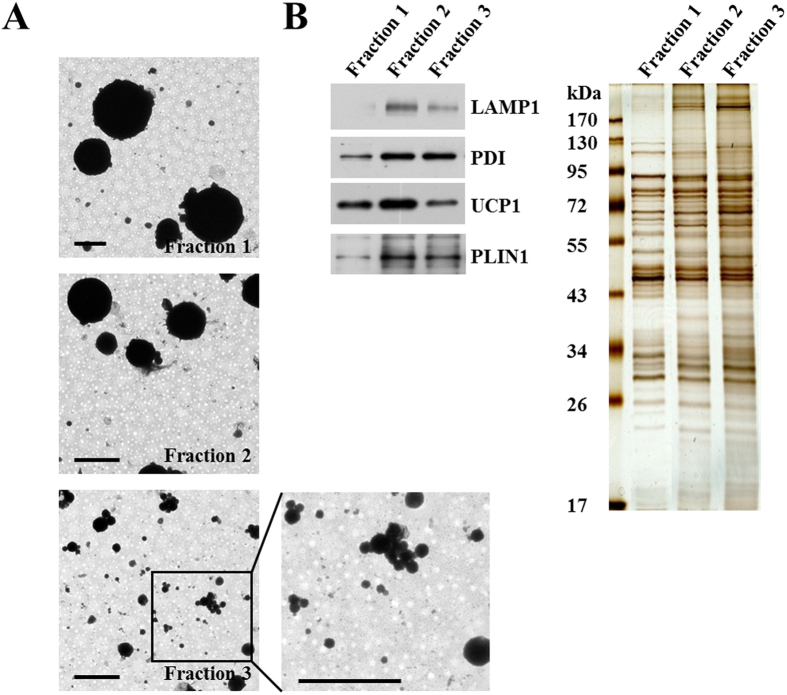
Separation of lipid droplet subpopulations from mouse brown adipose tissue by size. LDs from mouse BAT were fractionated by size using differential centrifugation (Materials and Methods). Four LD fractions were collected. (**A**) EM to determine LD size. The four LD fractions were processed for positive staining and the sizes of the LDs were analyzed by EM. Bar = 2 μm. (**B**) Protein profile of the subpopulations. Proteins of LD subpopulations were extracted with chloroform:acetone (300 μl:700 μl) and analyzed by Western blotting with the indicated antibodies and silver staining (equal amount of protein per lane).

We also adapted the method to brown adipose tissue. As with the other starting materials, the LDs from BAT were separated into LDs of different sizes (Fig. 5A). Fraction 1 contained LDs more than 4 microns while Fraction 3 was consisted of LDs less than 1 micron. The fractions exhibited different connections with ER or lysosomes (Fig. 5B). Interestingly, unlike the CHO K2 or liver LD subpopulations, which had similar quantities of ADRP, the LD fractions from BAT differed dramatically in the content of Perilipin 1 (PLIN1). Furthermore, the LD fractions had different quantities of UCP1, a mitochondrial inner membrane protein as well as a marker of activated mitochondria. Together the data show the method can fractionate LDs in BAT into different fractions with various sizes and functions.

### Lipid droplets nearly depleted of ER are able to incorporate free fatty acids into phospholipids and TAG

LDs have been shown to have lipid synthetic activity *in vitro*^1^. However, since most routinely isolated LDs contain substantial associated ER membrane, the role of LDs themselves in lipid synthesis remains debatable. Using this method we are able to isolate a sub-population of LDs from CHO K2 cells (Fraction 3) that contain triacylglycerol and phospholipid synthetic enzymes, but are nearly depleted in adherent organelles (Fig. 2D). Therefore, this permits an excellent test of the native lipid synthetic capacity of LDs. Using radio-labeled precursors, we tested the ability of Fraction 3 LDs in CHO K2 cells to synthesize lipids *in vitro*.

**Figure 6:**
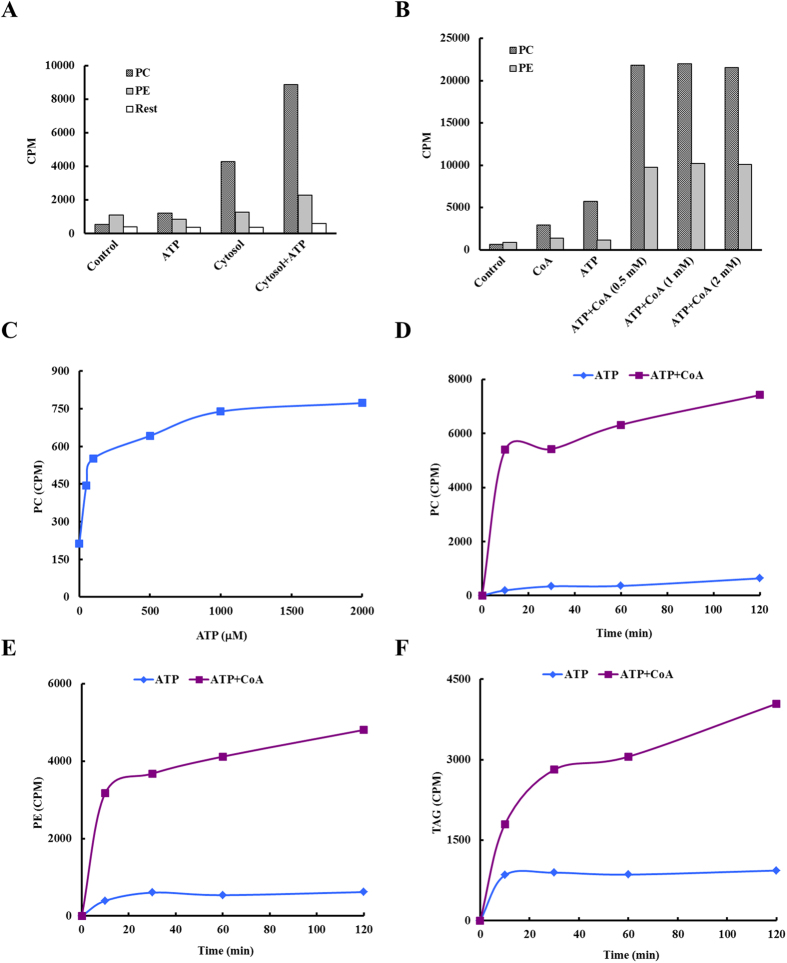
Fatty acid incorporation in ER-reduced lipid droplet subpopulations. LDs and cytosol were isolated from CHO K2 cells, and the LDs were further separated into three subpopulations using differential centrifugation (Materials and Methods). Fraction 3, the LD subpopulation nearly depleted of ER, was used for an *in vitro* fatty acid incorporation assay. The faction 3 LDs had first been incubated with [^3^H]OA and unlabeled OA at 4 °C for 6 h. (**A**) Requirement of ATP and cytosol for fatty acid incorporation. The OA-associated LDs were then aliquoted and incubated with 2 mM ATP alone or 100 μl cytosol in the presence or absence of 2 mM ATP at 37 °C for 2 h. (**B**) Requirement of ATP and CoA for fatty acid incorporation. The OA-associated LDs were then aliquoted and incubated with 2 mM CoA alone or the indicated concentrations of CoA (mM) in the presence or absence of 2 mM ATP at 37 °C for 2 h. (**C**–**F**) Time-dependent fatty acid incorporation. The OA-associated LDs were then aliquoted and incubated with the indicated concentration of ATP (μM) at 37 °C for 2 h in the presence of 100 μl cytosol (**C**), or with 2 mM ATP in the presence or absence of 2 mM CoA at 37 °C for the indicated times (**D**–**F**). After incubation, LDs were re-isolated, washed 3 times with Buffer B, and lipids extracted with acetone:chloroform (2:1, v/v) and separated using TLC. The bands of phosphatidylcholine (PC), phosphatidylethanolamine (PE), and triacylglycerol (TAG) were scraped from TLC plate and counted with scintillation fluid.

To assay for lipid synthetic activity, radio-labeled oleic acid (OA) in ethanol was dried under a stream of nitrogen. LDs were added to the tube and incubated to allow incorporation. Then, ATP and isolated cytosol were added and the reaction was incubated at 37 °C. After incubation, LDs were re-isolated and washed by centrifugation and the lipids were extracted and analyzed by thin layer chromatography (TLC). Bands corresponding to lipid standards were collected and the radioactivity was counted. The results presented in Fig. 6A demonstrate that LDs nearly depleted of ER are capable of incorporating fatty acids into phospholipids *in vitro* in a cytosol dependent manner. Then, using the same assay, we were able to determine that Coenzyme A (CoA) was the factor in cytosol required for the activity. Thus CoA could substitute for cytosol in supporting *in vitro* lipid synthesis (Fig. 6B). Next, the optimal ATP concentration required to support the reaction was determined (Fig. 6C).

Once optimal conditions to support lipid synthesis had been determined, the kinetics of synthesis of phosphatidylcholine (PC), phosphatidylethanolamine (PE), and TAG were measured (Fig. 6D–F). The incorporation of [^3^H]OA had biphasic kinetics with an initial rapid phase lasting about 15 minutes and a slower linear incorporation phase. These results indicate that the LD-associated lipid synthetic enzymes were active and LDs are one site of lipid synthesis in cells.

## Discussion

The low density of LDs makes them easy to be separated from other membranous structures using centrifugation. However, LDs have been demonstrated to have direct contact with other organelles, especially the ER. ER marker proteins and non-LD membrane structures are routinely detected in preparations of purified LDs. Previously, Jacquier *et al*. found that LDs are functionally connected to the ER membrane, with this connection allowing for the efficient partitioning of membrane proteins between the two compartments^11^. Our finding in Fig. 1 shows that ER lumenal proteins Bip was protected from trypsin digestion of a LD preparation, further suggesting that the tight association between LDs and intact ER could not be separated under such condition.

The study of LDs is complicated by their close association with other organelles and their heterogeneity in size. Therefore, isolation and separation of LDs by size and by their association with other organelles are very essential for study of the organelle. Using differential centrifugation of a density gradient, we were able to separate purified LDs into three fractions with different droplet sizes, which differed in the association with other membrane structures (Fig. 2). This method is rapid, simple and reproducible. Furthermore, this method can also be used with minor adaptations to separate subpopulation of LDs from other sources such as Huh7 cells, mouse liver and brown adipose tissue (Figs 3, 4, 5). Besides, as indicated by the lipid profiles of various LDs from mouse liver and CHO K2 (Fig. 4), the resulting distinct LD fractions through this method probably attributes to LD size rather than LD lipid composition.

The LD fraction containing the smaller LDs was largely free of contamination by membranes and proteins from other organelles (Figs 2A,B and 4A,C), which is in agreement with previous studies that ER membranes interact primarily with large LDs *in vivo*^10^,^39^. It should be noted, however, that this phenotype by using this method may not be the case for ‘fatty’ tissues (such as BAT, white adipose tissue, fatty liver, fatty heart), in which supersized LDs fill a cell, even though LDs from BAT could also be separated into different fractions with distinct sizes (Fig. 5). Besides, Blouin *et al*. found that caveolin-1-coated LDs were much larger than caveolin-1-devoid LDs *in vivo* by confocal study^47^, which is consistent with our current *in vitro* result (Fig. 2B). Also for Huh7 cells, different sizes of LDs with distinct interactions with lysosome and protein profiles were separated by this method (Fig. 3). According to our results, Fraction 4 from Huh7 cells contained very small LDs and most of them were surrounded by debris (Fig. 3). Biochemical analysis indicated they were abundant in ER proteins. These data agree with the current point that LDs come from ER membrane and the newly-formed small LDs should be ER-connected^11^,^40^. Another possibility is that the real small LDs may be connected with ER membrane thus never be able to float to the top of gradient.

We further observed that differences in LD size were associated with distinct functions as suggested by their differential recruitment of cytosolic proteins (Fig. 2C). LDs of different sizes have different surface curvatures, which may require specific phospholipids and protein compositions to maintain their structures. These differences in lipid and protein compositions may also be associated with distinct functions.

A great number of lipid metabolic enzymes have been reported to localize to LDs. This was also the case in the current study and most of the enzymes were found to have a similar abundance on all LD subpopulations. However, two long-chain acyl-CoA synthetases, ACSL3 and ACSL4, were heterogeneously distributed among the LD populations (Fig. 2D). Knockdown of the two enzymes in hepatocytes showed totally different effects on the incorporation of fatty acids into phosphatidylcholine and ultimately, the assembly of very low density lipoprotein^48^. Different ACSL isoforms can direct fatty acids and their synthetic products towards distinct metabolic pathways^49^. All suggest that the subpopulations of LDs that we obtained were functionally distinct.

As shown in Fig. 2B,D, small G-proteins including Rho and Rab family proteins were enriched in the smaller LDs in Fraction 3. Small G-proteins are very active in cellular processes, including cell proliferation, cytoskeletal dynamics, and vesicle transport. The preferential distribution of these G-proteins to small LDs indicates that the small LDs in a cell may be more active in those cellular functions than larger LDs. Rab 18 was among the proteins enriched in the small LD population. It has been reported that Rab 18 labeled a distinct subset of LDs in both adipocytes and non-adipocytes and the regulation of the recruitment was shown to rely on the metabolic state of the LDs^9^. However, the exact molecular mechanism underlining how Rab 18 was recruited to specific LD populations was unclear. Our method for isolating LDs enriched or depleted in Rab 18 may provide a powerful tool to address the mechanism, perhaps leading to a better understanding of the mechanisms underlying metabolic diseases.

The SDR family proteins are present in all living organisms and have important roles in the control of cellular metabolism^50^,^51^. Our recent results showed that 17βHSD13, localized on LDs, is a pathogenic protein in nonalcoholic fatty liver disease^52^. The enrichment of SDR family proteins on the cellular organelle-depleted LD subpopulation suggests that SDR proteins are LD-residents (Fig. 2D). In support of this notion, proteomic studies on LDs from almost all sources, such as liver^52^, skeletal muscle^5^, brown adipose tissue^53^, plant^54^, *C. elegans*^55^, *Drosophila*^56^, yeast^57^, and bacteria^20^, identified SDR proteins on LDs. For example, in *C. elegans*, a short-chain dehydrogenase DHS-3 is one of the main proteins of LDs and it exclusively localizes on LDs^55^.

Using the method we developed the LDs isolated from mouse liver could be separated into fractions relatively enriched in CE or TAG (Fig. 4). This finding provides the groundwork to search for a marker protein to differentiate these populations, which could facilitate research into the metabolism of TAG and CE. This may provide valuable insights into the involvement of the liver in metabolic diseases.

In our experiments with BAT, we found that the mitochondrial inner membrane protein UCP1 was well represented in all three LD fractions, making it quite distinct from ER and lysosomal marker proteins. This indicates that LDs and mitochondria are especially tightly associated in this tissue type (Fig. 5). This result is consistent with our recent finding that BAT LDs are physiologically bound to mitochondria. Even when treated with high concentration salt, detergent or proteinase, BAT LDs still tightly associated with intact mitochondria^53^. The varying proportional representation of different mitochondrial proteins in the three LD fractions suggests that there may be important functional distinctions among the mitochondria that are bound to different LD populations. This is an intriguing possibility that could deserve further investigation.

The ER is a site of lipid ester synthesis^15^,^26^, but LDs have also recently been proposed to be a synthetic site for a number of lipids^2^. There have been several reports on lipid synthesis or enzymatic activities in isolated LDs^26^,^27^,^28^,^29^,^30^. However, in those studies, fatty acids dissolved in ethanol were added directly to the reactions, a procedure which results in a limited amount of this hydrophobic substrate being available for the reaction taking place in an aqueous environment. Our experiments using a method in which LDs recruited fatty acids from the reaction tube prior to initiating the reaction and allowing LDs to incorporate the substrate directly. Using this method we determined that only ATP and CoA were required to support lipid synthesis in this system (Fig. 6B).

The fatty acid incorporation measured in these *in vitro* reactions exhibited saturation kinetics, supporting the conclusion that enzymes localized to LDs are responsible for this reaction and suggesting our assay is effective. The reaction exhibited biphasic kinetics (Fig. 6) suggesting that a pre-existing pool of lysophospholipids and diacylglycerol were present on the LDs that support the initial, rapid phase. This conclusion is in agreement with our previous report^37^. Exhaustion of these intermediates results in a dramatic slowing of the reaction as an earlier synthetic reaction becomes the rate limiting step. If true, this is consistent with the LD as a lipid factory, providing not just the raw materials, but also the enzymatic capacity to actively participate in lipid synthesis.

It has been difficult to reach a definitive conclusion regarding the role of LDs in lipid synthesis due to the presence of adherent ER membrane in standard preparations. In this study, we found that the distinct LD populations showed no obvious differences in the abundance of lipid metabolic enzymes (Fig. 2D). However, we were able to prepare a LD fraction lacking any significant contamination by ER or other organelle membrane. These LDs were able to incorporate radio-labeled fatty acids into phospholipids and neutral lipids (Fig. 6). It is hard to exclude the existence of trace amount of ER in this fraction; however, the obvious decrease of many ER proteins in this fraction suggests its self-sustaining state (Fig. 2 and MS data). Acyl-CoA:diacylglycerol acyltransferase (DGAT) is the key enzyme catalyzing the synthesis of TAG in eukaryotic organisms. Interestingly, MS data showed there were no DGATs in this fraction. Impressively, we found that after knockout of both DGATs in C2C12 cells about 10% TAG was still in the cells compared to wild type (unpublished data). These data suggest that LDs contain another acyltransferase activity for triacylglycerol synthesis. Recently, McFie *et al*. found that when the localization of DGAT2 was disrupted, DGAT2 was still able to promote TAG synthesis without interaction with the ER. They concluded that TAG synthesis and LD formation do not require the presence of DGAT2 in the ER in HEK-293T and COS-7 cells^58^. This finding also indicates that the LD may be another important site for lipid metabolism.

A prominent hypothesis holds that LDs increase in size by fusion of smaller LDs^59^. However, our discovery of the incorporation of fatty acids into lipids on the LD suggests that LDs may be able to grow through *in situ* lipid synthesis, although more evidence will be required to reach this conclusion. Interestingly, Walther’s group also found that LDs grew when TAG synthesis enzymes relocalized from ER to LDs^40^.

Our method successfully separates LDs by size and produces fractions that vary in their association with other organelle membranes. The method can be applied successfully to LDs from several tissue and cell sources. The biochemical isolation of LDs with different sizes may help to elucidate any distinct roles of large and small LDs in biological processes. The method will also permit the determination of the protein and lipid composition of LDs almost without interference by ER or other organelle components. This will facilitate investigation into the unique biology of LDs as well as the relationship between LD and other organelles. Furthermore, our method for studying LD-mediated lipid synthesis *in vitro* is more effective and efficient than previously described approaches. Using our purification process, we have provided clear evidence that LDs have a native capacity to synthesize lipids independent of the ER or other organelles.

## Materials and Methods

### Materials

Calf serum and DMEM were from Hyclone. Penicillin-Streptomycin solution (100×) and Colloidal Blue Staining Kit were from Life Technologies. ECL Western Blotting Substrate was from Thermo Scientific.

ADRP monoclonal antibody (mAb) was from Dr. Ginette Serrero’s group. Cav-1 polyclonal antibody (pAb), mAbs against Rab 5, Bip, p62, Rab 11, PDI, Rab 4 and Tim23 were purchased from BD Biosciences. pAbs against Rab 18 and Sec 61, mAbs against Porin and GAPDH were from Millipore. ARF1 mAb was from Santa Cruz. βCOP mAb was from Sigma. Tom20 pAb and RhoA mAb were from Santa Cruz Biotech. 17βHSD11 mAb and CPT1a mAb were from abcam. 17βHSD12 pAb was from Thermo Scientific. pAb against DHRS3, ACSL4, GPAT4, LPCAT1, Reep5 and CGI-58 were from ABclonal Technology. pAb against ACSL3 was from Proteintech. pAb against ATGL and mAb against LAMP1 were from Cell Signaling Technology. UCP1 mAb was a gift from Dr. Peng Li. PLIN1 pAb was a gift from Dr. Guoheng Xu.

GTPγs was from Millipore. Proline, PMSF, oleic acid, lipid standard, ATP, Coenzyme A sodium salt sodium salt hydrate and tannic acid were from Sigma. 25% glutaraldehyde solution (EM grade), Embed 812 kit, uranyl acetate and lead citrate were from Electron Microscopy Sciences. Osmium tetraoxide (EM grade) was purchased from Nakalai Tesque. Formvar was from BDH Chemicals. [9,10-^3^H(N)]-oleic acid (OA) (5 mCi/ml) was from PerkinElmer Life Sciences.

### Culture of CHO K2 cells

CHO K2 cells were cultured in 150 mm dishes in high glucose DMEM containing 10% calf serum, 40 μg/ml proline, 100 U/ml penicillin and 100 μg/ml streptomycin at 37 °C with 5% CO_2_. The cells were cultured until confluent and the culture medium was refreshed 12 h before isolation of LDs.

### Isolation of total lipid droplets from CHO K2 cells

Total LDs were isolated from CHO K2 cells according to the protocol previously described^46^. Briefly, cells from twenty 150 mm dishes were washed twice with ice-cold PBS and were then collected by scraping them into ice-cold PBS. The cells were pelleted by centrifugation at 1,000 *g* for 10 min at 4 °C, resuspended in Buffer A (20 mM tricine, 250 mM sucrose, pH 7.8) containing 0.5 mM PMSF and kept on ice for 20 min. The cell suspension was pressurized to 35 bar with N_2_ and held on ice for 15 minutes, followed by homogenization by cavitation. The homogenate was centrifuged at 3,000 *g* for 10 min at 4 °C and the supernatant was transferred into SW 40 Ti tubes. Then 2 ml of Buffer B (20 mM HEPES, 100 mM KCl, 2 mM MgCl_2_, pH 7.4) was loaded onto the top of the supernatant and the gradient was centrifuged at 182,000 *g* for 1 h at 4 °C. The top band was collected to a 1.5 ml microcentrifuge tube, which was the Total LDs for further subpopulation. Part of the preparation was centrifuged at 20,000 *g* for 3 min at 4 °C. The solution underneath the white band was removed and the LDs were resuspended in 200 μl of Buffer B, and then centrifuged again. This step was repeated 3 to 4 times until no pellet was visualized. The resulting droplet fraction was defined as original LDs.

### Fractionation of total lipid droplets from CHO K2 cells

1 ml of total LD solution (~200 μg protein) was mixed with 1 ml of 2.5 M sucrose in a SW 40 Ti tube by gentle vortexing. The LD solution was overlaid sequentially with 4 ml of 625 mM sucrose in Buffer B, 2 ml of 313 mM sucrose in Buffer B, and 4 ml Buffer B. The gradient was centrifuged at 500 *g* for 20 min. After centrifugation, fractions were collected from the top of the gradient, 1 ml each, and proteins of these fractions were precipitated using 7.2% trichloroacetic acid and blotted by indicated antibodies.

### Subpopulation of total lipid droplets from CHO K2 cells

0.8 ml of 2.5 M sucrose was mixed with 1 ml of total LDs (~200 μg protein) and then adjusted to a final sucrose concentration of 500 mM with Buffer B. The LD solution (4 ml) was transferred to a SW 40 Ti tube and overlaid with 4 ml of 250 mM sucrose in Buffer B and 4 ml Buffer B, sequentially. The gradient was centrifuged at 500 *g* for 40 min. The white band on the top was carefully collected in 100 μl using a 200 μl pipette and transferred into a 1.5 ml microcentrifuge tube (Fraction 1). Then 100 μl Buffer B was added to balance the SW 40 Ti tube and the gradient was centrifuged again at 2,000 *g* for 20 min and then at 8,000 *g* for 20 min at 4 °C successively. The top bands were collected as Fraction 2 and 3 respectively. The collected three LD preparations were centrifuged at 20,000 *g* at 4 °C for 3 min. The underlying solution was removed with a gel-loading tip. The resulting preparations were ready to be analyzed.

### Isolation of cytosol

During purification of the total LDs, 1 ml of the solution from the middle of the gradient in the SW 40 Ti tube was taken and then centrifuged in a TLA100.3 rotor at 270,000 *g* for 1 h at 4 °C. The supernatant was collected and was used directly without freezing.

### Trypsin protection assay

Total LD preparation (50 μl, equivalent to 10 μg protein) was incubated with 0, 0.0125%, 0.025% or 0.05% of trypsin at 37 °C for 10 min, 90 min or 120 min. Reactions were vortexed slightly every 10 min. After the incubation the reactions were centrifuged at 20,000 *g* for 3 min and the buffer underneath was removed. Then LDs were collected and washed with Buffer B 3 times and LD proteins were analyzed by Western blotting with indicated antibodies.

### Treatment with high concentration salt

50 μl of total LDs (~10 μg protein) were incubated in 200 μl Buffer B alone or Buffer B containing 2 M NaCl. Samples were kept at 4 °C for 30 min, vortexed gently every 10 min, and then centrifuged at 20,000 *g* for 3 min. The LD layer was collected and proteins were precipitated with acetone and subjected to Western blotting. The buffer underneath LDs was also collected and mixed with 5 × SDS sample buffer (to a concentration of 2 × SDS sample buffer) then analyzed by Western blotting.

### Recruitment assay

50 μl of LD preparation (~10 μg protein) was incubated in 100 μl Buffer B alone or in 100 μl of cytosol (~80 μg protein) in Buffer B plus 1 mM GTPγs for 1 h at 37 °C. The reaction system was vortexed gently every 10 min. LDs were then re-isolated by centrifugation at 20,000 *g* for 3 min and washed with Buffer B 5 times. LD proteins were analyzed by Western blotting with indicated antibodies.

### Protein preparation, Western blotting, and lipid analysis

Following the removal of the suspension buffer, LDs were treated with chloroform-acetone (300 μl:700 μl) at room temperature. The tubes were strongly vortexed and then centrifuged at 20,000 *g* for 10 min. The pelleted proteins were dissolved in 2 × SDS sample buffer and separated with 10% SDS-PAGE. The proteins were then either analyzed by Colloidal Blue staining or silver staining or transferred to a PVDF membrane, blotted with indicated antibodies and detected with the ECL system. The organic supernatant was transferred to a new tube and evaporated under a stream of nitrogen and analyzed with TLC, developing solvent of hexane-diethyl ether-acetic acid (80:20:1, v/v/v). TLC plates were stained in iodine vapor after drying at room temperature.

### Isolation and subpopulation of lipid droplets from Huh7 cells

Similar to CHO K2 cells, confluent Huh 7 cells from forty 100 mm dishes were washed, collected and resuspended in Buffer A as described above. After 20 min on ice, the cells were homogenised by cavitation. The homogenate was centrifuged at 500 *g* for 5 min at 4 °C to remove the cell debris. The resulting supernatant was loaded into a SW 40 Ti tube with around 2 ml Buffer B on the top and then was centrifuged at 500 *g* for 20 min. The white band was collected with a 200 μl pipette tip. Subsequently, the tube was centrifuged at 2000 *g* for 20 min, 8000 *g* for 20 min and 182,000 *g* for 40 min. Each top white band was collected and the one collected after the last centrifugation was kept and named Fraction 4. The other three bands were mixed together. This mixture was transferred into a SW 40 Ti tube and 2.5 M sucrose was added to a final concentration of 500 mM in Buffer B, 4 ml in total. 4 ml of 250 mM sucrose in Buffer B and 4 ml Buffer B were loaded sequentially. This gradient was centrifuged at 500 *g* for 20 min, 2000 *g* for 20 min and 8000 *g* for 20 min sequentially. After each centrifugation, LDs were carefully collected in a minimal volume with a 200 μl pipette tip and an equal volume of Buffer B was added to the top. The three fractions collected were designated as Fraction 1, 2 and 3 respectively.

### Isolation and subpopulation of lipid droplets from mouse Liver

Mice were purchase from Vital River Laboratories. All animal procedures were approved by the Animal Care and Use Committee of the Institute of Biophysics, Chinese Academy of Sciences under the permission number SCXK (SPF2009_111). All experimental methods were carried out in accordance with the NIH Guide for the Care and Use of Laboratory Animals (Eighth Edition). Three 10-week-old male C57/L6 mice were sacrificed by cervical dislocation. Livers were collected and rinsed by ice-cold PBS twice with the gall bladders discarded. The connective tissues were carefully removed. The livers were transferred into 20 ml ice-cold Buffer A containing 0.5 mM PMSF and then sliced into small pieces around 1 mm^3^ by tweezers. The prepared liver tissues were homogenized with a loose-fitting glass-Teflon Dounce homogenizer by 15–20 passes on ice.

The homogenate was centrifuged at 500 *g* for 5 min to remove tissue debris and blood cells. The supernatant was transferred to a new tube. Care was taken to collect any white or pale yellow material at the top of the tube. Then 8.5 ml of the supernatant was loaded into a SW 40 Ti tube. 3 ml of Buffer B was overlaid on the top carefully without disturbing the supernatant. The gradient was centrifuged at 500 *g* for 20 min at 4 °C. The white band on the top was collected in a minimal volume into a 1.5 ml microcentrifuge tube (Fraction 1) with a 200 μl pipette tip.

An equal volume of Buffer B was layered on the top of the gradient to balance the tube and the tube was centrifuged again at 2,000 *g* for 20 min at 4 °C, followed by collection of the droplets at the top of the gradient (Fraction 2). LDs were collected again after centrifugation at 8,000 *g* for 20 min (Fraction 3) and 182,000 *g* for 20 min (Fraction 4). The four collected LD preparations were centrifuged at 4 °C for 4 min at the force used to acquire them. The underlying solution and pellet was removed using a 200 μl gel loading pipette tip. To the remaining LDs 200 μl Buffer B was added and the LDs were gently suspended. The steps above were repeated until no pellet could be observed following centrifugation.

### Isolation and subpopulation of lipid droplets from mouse brown adipose tissue

One 8-week-old male C57/L6 mice was sacrificed by cervical dislocation. Brown adipose tissue was carefully dissected from between the scapulae, ensuring it was free of adhesive white fat tissue or other connective tissue. The tissues were rinsed with ice-cold PBS and transferred into 2.5 ml ice-cold Buffer A with 0.5 mM PMSF, where they were minced into 2–3 mm^3^ pieces with dissecting scissors. To homogenize, the minced tissues were passed through a stainless steel sieve (200 mesh, 70 micron) with the aid of a syringe plunger into a 1.5 ml microcentrifuge tube. The tube was kept on ice for 20 min. After that, the sample was centrifuged at 500 *g* at 4 °C for 2 min. The white band on the top was collected in a smallest possible volume of buffer into a 1.5 ml microcentrifuge tube (Fraction 1) using a 200 μl pipette tip. Then the sample was centrifuged at 2,000 *g* at 4 °C for 15 min and again at 20,000 *g* at 4 °C for 30 min successively. Following each centrifugation step, the white band at the top of the tube was collected as Fractions 2 and 3, respectively. The three collected LD preparations were centrifuged respectively at 4 °C for 3 min at the force used to acquire them. The underlying solution and pellet were removed and discarded with a gel-loading tip. 200 μl Buffer B was added and LDs and they were gently suspended. The washing step was repeated until no pellet could be observed.

### Preparation for transmission electron microscopy study

Isolated LDs suspended in 100 μl Buffer B were mixed with an equal volume of 2% glutaraldehyde in 0.1 M PB (pH 7.4) and then incubated for 0.5 h at room temperature. Then an equal volume of 2% OsO_4_ was added followed by incubation for 0.5 h at room temperature. Then fixed LDs were collected by centrifugation and processed for dehydration and infiltration and were finally embedded with Epon (Embed 812). Then, 70 nm sections were prepared with a Leica EM UC6 Ultramicrotome. The sections were stained with 4% uranyl acetate for 15 min at room temperature and then with 2% lead citrate for 5 min. The samples were viewed with a JOEL 1200 or a Tecnai Spirit electron microscope.

Huh7 cells were rinsed with 0.1 M PB and then fixed in 2.5% glutaraldehyde in 0.1 M PB (pH 7.4) at room temperature for 2 h. After that, the cells were scraped into a 1.5 ml microcentrifuge tube and post-fixed with 1% (w/v) osmium tetraoxide (with 1% potassium ferrocyanide) for 1.5 h at room temperature. The cells were then dehydrated, embedded and treated as described above. The ultra-thin sections were viewed with Tecnai Spirit electron microscope.

The size of LDs was also examined by positive staining. A Formvar-covered copper grid was used to load LDs, with the grid placed on a drop of isolated LD suspension for 1 min. Then the grid was then placed on a drop of 2.5% glutaraldehyde solution (0.1 M PB, pH 7.4) for 10 min and subsequently on a drop of 2% osmium tetraoxide solution (0.1 M PB, pH 7.4) for 10 min to fix the LDs. The LDs were then stained with 0.1% tannic acid for 10 min and 2% uranyl acetate for 10 min. After each step, the grid was washed 3 times with deionized water, 1 min each time. The grids were finally viewed with Tecnai Spirit electron microscope.

### Preparation of oleic acid

Oleic acid (OA) was mixed with ethanol to a concentration of 100 mM. Then the mixture was sonicated on ice at 200 W, 10 s on and 3 s off pulses, until the solution was milky and homogenous. The prepared OA stocks were kept at 4 °C.

### *In vitro* assay of fatty acid incorporation by lipid droplets

2 μl of [^3^H]OA (10 μCi) and 1 μl of 100 mM OA in ethanol were mixed thoroughly in a microcentrifuge tube and then dried under N_2_ at room temperature. Then LDs (~10 μg protein) were added to the tube and incubated at 4 °C for 6 h. Then, depending on the experiment, ATP, cytosol or CoA was added to the tube and incubated at 37 °C for various times. After the incubation, LDs were re-isolated by centrifugation at 20,000 *g* for 3 min and washed with Buffer B 3 times. Total lipids were extracted with acetone-chloroform (2:1, v/v) and analyzed by TLC with a developing solvent of chloroform-acetone-methanol-acetic acid-water (80:40:30:20:10, v/v/v/v/v) for phospholipids, and hexane-diethyl ether-acetic acid (80:20:1, v/v/v) for neutral lipids. TLC plates were stained in iodine vapor after drying at room temperature. Bands corresponding to lipid standard separated on the same plate were scraped, collected and the radioactivity was measured using a Perkin Elmer scintillation counter.

## Additional Information

**How to cite this article**: Zhang, S. *et al*. Morphologically and Functionally Distinct Lipid Droplet Subpopulations. *Sci. Rep*. **6**, 29539; doi: 10.1038/srep29539 (2016).

## Supplementary Material

Supplementary Information
