# Supplementary material for: Morphologically and Functionally Distinct Lipid Droplet Subpopulations
Source: Sci Rep. 2016 Jul 8;6:29539. doi: 10.1038/srep29539 (PMC4937419; doi:10.1038/srep29539)
Supplement: Supplementary Information [file srep29539-s1.pdf]

Supplemental Information for

Morphologically and Functionally Distinct Lipid Droplet Subpopulations

Shuyan Zhang[a §], Yang Wang[a §], Liujuan Cui[a,b], Yaqin Deng[a,c], Shimeng Xu[a,c], Jinhai Yu[a,c], Simon Cichello[d], Ginette Serrero[e], Yunshu Ying[f], and Pingsheng Liu[a]*

Materials and Methods

Mass spectrometry (MS) analysis of lipid droplet proteins from CHO K2 Cells

LD proteins were dissolved at a concentration of 10 mg/ml in 100 mM Tris (pH 7.8), containing 8 M urea. The proteins were then reduced with 10 mM DTT for 1 h at RT. 40 mM iodoacetamide was added to alkylate the protein mixture for 1 h at RT in the dark. 40 mM DTT was used to consume the unreacted iodoacetamide for 1 h at RT. Then the urea concentration was reduced to less than 2 M with super clean water. Trypsin was then added at an enzyme:substrate ratio of 1:50 (w/w) and the reaction mixture was incubated overnight in 37 ℃ water bath. 10% formic acid was added to stop the reaction, making pH less than 4. The resulting peptide solution was then subjected to nano-LC-ESI-LTQ MS/MS analysis according to the protocol previously described[1]. Briefly, a C_{18} trap column was used and the mass spectrometer was operated in data-dependent mode and set at an initial 400-2000 *m*/*z* scan range, followed by five MS/MS scans. The MS/MS data were searched against the NCBI Refseq mouse database that was released on Jan. 3, 2011. BioWorks search parameters were set as follows: enzyme, trypsin; precursor mass tolerance, 3 Da; fragment ion mass tolerance, 1 Da. The fixed modification was set for carboxyamidomethylation of cysteine (Cys +57.02 Da). The search results were filtered as follows: Xcorr (+1) ≥ 1.5, Xcorr (+2) ≥ 2.0, Xcorr (+3) ≥ 2.5; Delta CN ≥ 0.08; Sp score ≥ 500; RSp ≤ 5; Number of distinct peptides ≥ 2.

Supplemental Table 1. Proteins in various LD subpopulations from CHO K2 cells identified by MS.

Sub-cellular location/Function	Proteins	Aliases/Short name	Peptide number in LD Fractions			GI No.
			1	2	3	
**Perilipin family**						
	perilipin-2	ADRP	7	7	7	116235489
**Lipid metabolism**						
	long-chain-fatty-acid--CoA ligase 4	LACS 4	45	52	40	341940183
	long-chain-fatty-acid--CoA ligase 3	LACS 3	31	31	29	341940182
	patatin-like phospholipase domain-containing protein 2	ATGL	14	10	12	81896337
	comparative gene identification-58	CGI-58	6	10	8	13385690
	lysophospholipid acyltransferase LPCAT4	1-AGPAT 7	6	9	7	46402175
	squalene monooxygenase	SE	5	3	3	6678127
	NADH-cytochrome b5 reductase 3	B5R	8	9	7	19745150
	retinol dehydrogenase 10	Rdh10	4	4	3	25141231
	saccharopine dehydrogenase-like oxidoreductase	CGI-49	7	6	6	73919295
	estradiol 17-beta-dehydrogenase 11	17bHSD11	5	7	6	16716597
	sterol-4-alpha-carboxylate 3-dehydrogenase, decarboxylating	NSDHL	4	2	2	31982437
	glycerol-3-phosphate acyltransferase 6 precursor	GPAT6	2	7	6	30520301
	acetyl-CoA carboxylase 1	ACC1	2	2	3	125656173
	lanosterol synthase	OSC	2	3	4	22122469
	hormone-sensitive lipase	HSL	3	0	3	85690846
	dehydrogenase/reductase SDR family member 1	DHRS1	4	0	4	31980844
	short-chain dehydrogenase/reductase 3	retSDR1	2	0	0	47115587

glycerol-3-phosphate acyltransferase 3	GPAT3	2	0	0	27370046
long-chain-fatty-acid--CoA ligase 1	LACS 1	0	2	0	31560705
3-keto-steroid reductase	17bHSD7	0	0	2	87162470
**ER**					
transitional endoplasmic reticulum ATPase	VCP	19	15	12	225543319
78 kDa glucose-regulated protein	Bip	19	12	9	2506545
protein disulfide-isomerase A3 precursor	ERp60	5	6	2	112293264
sarcoplasmic/endoplasmic reticulum calcium ATPase 2	SERCA2	6	2	3	12643614
leucine-rich repeat-containing protein 59	LRC59	5	3	3	19527026
ancient ubiquitous protein 1	AUP1	4	5	2	90403601
nogo-B receptor precursor	NgBR	2	4	3	13384840
endoplasmin precursor	GRP-94	8	8	0	6755863
cytoskeleton-associated protein 4	Climp-63	5	2	0	62526118
dolichyl-phosphate beta-glucosyltransferase	ALG5	3	3	0	21728372
serpin H1 precursor	serpin H1	2	2	0	161353502
protein disulfide-isomerase A6 precursor	ERp5	4	0	2	58037267
protein disulfide-isomerase precursor	PDI	4	0	0	42415475
dolichyl-diphosphooligosaccharide--protein glycosyltransferase subunit 2	RPN-II	3	0	0	34996495
hypoxia up-regulated protein 1 precursor	GRP-170	2	0	0	157951706
membrane-associated progesterone receptor component 1	mPR	2	0	0	31980806
calreticulin precursor	CRP55	0	2	0	6680836
**Mitochondria**					
ATP synthase subunit alpha, mitochondrial precursor	ATPsyn-alpha	16	5	5	6680748

ATP synthase subunit beta, mitochondrial precursor	ATPsyn-beta	12	6	7	31980648
ADP/ATP translocase 2	Ant2	5	6	4	22094075
prohibitin	prohibitin	3	3	3	6679299
phosphate carrier protein, mitochondrial precursor	PTP	2	2	2	19526818
prohibitin-2	prohibitin-2	4	2	0	126723336
sideroflexin-1	TCC	2	2	0	15147224
60 kDa heat shock protein, mitochondrial	HSP60	3	0	0	183396771
stAR-related lipid transfer protein 7, mitochondrial precursor	StARD7	2	0	0	151301231
protein NipSnap homolog 1	NipSnap1	2	0	0	6679066
**Membrane trafficking**					
Rab-18	Rab 18	13	11	11	30841008
Rab-14	Rab 14	10	11	12	18390323
Rab-7a	Rab 7a	8	11	12	148747526
Rab-1B	Rab 1b	8	11	12	21313162
Rab-1A	Rab 1a	5	11	8	6679587
Rab-2A	Rab 2a	7	8	7	10946940
Rab-5C	Rab 5	6	4	7	113866024
Rab-5B		2	2	0	48474314
Rab-31	Rab-22B	6	5	4	225579124
Rab-10	Rab 10	4	4	4	7710086
Rab-6	Rab 6	3	2	2	13638404
Rab-35	Rab 35	2	2	2	37718983
Rab-11B	Rab 11b	4	4	0	6679583
Rab-33B	Rab 33b	2	2	0	8394133
Rab-11A	Rab 11a	4	0	8	31980840

Rab-8A	Rab 8a	3	0	3	38372905
Rab-8B		2	0	0	27734154
Rab-3D		0	2	2	15042957
Rab-4A	Rab 21	2	0	0	171184402
Rab-21		2	0	0	33859751
Rab-7L1	Rab-7-like protein 1	0	2	0	229608951
Rab-34	Rab 34	0	0	3	20141805
Rab-22A		0	0	3	148747177
Rab-32		0	0	2	13385896
synaptic vesicle membrane protein VAT-1 homolog		6	8	9	33859662
ras-related protein Ral-A precursor	RalA	3	4	4	34328471
ras-related protein Rap-1b precursor	GTP-binding protein smg p21B	6	7	4	33859753
transmembrane emp24 domain-containing protein 10 precursor	S31I125	2	3	3	21312062
coatomer subunit beta	Beta-COP	3	2	0	15426055
charged multivesicular body protein 6	Vps20	0	2	2	147904547
GTPase NRas precursor	Transforming protein N-Ras	0	2	0	111154109
ras-related protein Ral-B precursor		0	0	4	11612509
**Ribosome**					
60S ribosomal protein L4		4	3	2	30794450
60S ribosomal protein L7a		3	2	2	7305443
60S ribosomal protein L27a		2	2	2	31560517
60S ribosomal protein L21		2	2	3	2500367
60S ribosomal protein L10		3	3	0	16418339
40S ribosomal protein S3		0	2	2	6755372

60S acidic ribosomal protein P0		2	0	0	6671569
60S ribosomal protein L18		2	0	0	83699424
40S ribosomal protein S13		2	0	0	13386034
60S ribosomal protein L24		2	0	0	18250296
60S ribosomal protein L30		0	3	0	6677783
40S ribosomal protein S8		0	2	0	6677813
60S ribosomal protein L7		0	2	0	31981515
40S ribosomal protein S2		0	2	0	46397787
60S ribosomal protein L23a		0	2	0	51338638
PREDICTED: 40S ribosomal protein SA-like		0	2	0	309264022
PREDICTED: 40S ribosomal protein S8-like		0	2	0	309266495
PREDICTED: 60S ribosomal protein L7a-like		0	2	0	82950040
60S ribosomal protein L8		0	0	2	6755358
60S ribosomal protein L5		0	0	2	23956082
PREDICTED: 60S ribosomal protein L5-like		0	0	2	309267832
**Nuclear proteins**					
histone H4		11	8	7	51317340
lamin-B1		7	7	9	188219589
histone H2A		4	4	5	30061357
transmembrane protein 43	Protein LUMA	4	3	4	21311891
histone H2B		3	5	5	160420310
histone H1.3		3	3	2	254588110
histone H3.3		2	0	2	55977063
prelamin-A/C	70 kDa lamin	3	3	0	85700428
heat shock cognate 71 kDa protein		0	2	0	31981690

thymocyte nuclear protein 1		0	2	0	21362343
transformation/transcription domain-associated protein		0	0	2	124486949
**Cytoskeleton**					
vimentin		9	12	12	31982755
annexin A2	PAP-IV	3	2	3	6996913
actin		6	7	4	6752954
tubulin beta-5 chain		12	7	7	7106439
tubulin alpha-1B chain		3	0	0	34740335
tubulin beta-4 chain		0	0	4	31981939
tubulin beta-2A chain		0	0	4	33859488
desmin		0	2	0	33563250
**Cytosolic proteins**					
heat shock protein HSP 90-beta		4	0	0	40556608
heat shock protein HSP 90-alpha		2	0	0	6754254
elongation factor 1-alpha 1	EF-1-alpha-1	3	4	5	126032329
polyadenylate-binding protein 1		2	0	0	31560656
**Protein degradation**					
FAS-associated factor 2	UBXD8	12	12	13	158533976
UBX domain-containing protein 4		3	5	5	85861252
ubiquitin-conjugating enzyme E2 G2		2	3	2	20806111
ubiquitin conjugation factor E4 A	UBE4A	2	2	0	167736371
ubiquitin-40S ribosomal protein S27a precursor	RPS27A	0	2	2	13195690

**Signal transduction**					
tumor protein D54	hD54, D52-like 2	6	6	8	31560247
calcium-binding protein p22		4	2	5	9790225
guanine nucleotide-binding protein G(I)/G(S)/G(T) subunit beta-2		3	3	2	13937391
methyltransferase-like protein 7A	AAM-B	2	3	3	33563290
transforming protein RhoA precursor	Rho A	2	3	3	31542143
rho-related GTP-binding protein RhoG precursor	Rho G	2	3	0	9625037
cell division control protein 42 homolog isoform 1 precursor		0	3	0	6753364
**Other**					
tumor protein p63-regulated gene 1-like protein		3	3	5	21312776
vacuolar protein sorting-associated protein 13C		4	3	0	122114537
uncharacterized protein LOC239673		2	0	3	269914154
polymerase I and transcript release factor	Cavin-1/PTRF	0	2	0	6679567
aladin	Aladin/Adracalin/AAAS	0	0	2	241982696

Supplemental Figure 1 Interaction between lipid droplets and other organelles in mouse liver.

Fractions 2 and 3 from mouse liver were isolated as described in the Materials and Methods. They were mixed first and then distributed into 6 aliquots. The isolated LDs were treated with trypsin at 37 ℃ for 60 min at the concentration (%, w/v) indicated. After incubation, LDs were re-isolated, and proteins were extracted and analyzed by silver staining and Western blotting with the indicated antibodies.

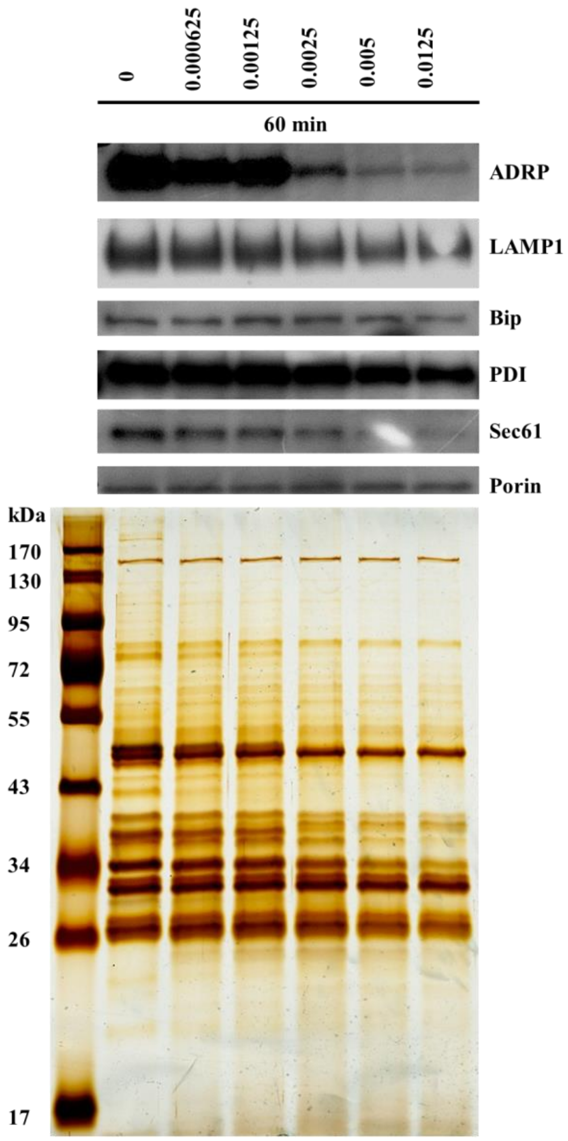

Supplemental Figure 2 Cross correlation analysis of proteins in different LD subpopulations from CHO K2.

Proteins from different LD fractions in CHO K2 cells were subjected to MS analysis respectively as described in the Materials and Methods. Proteins identified in MS of LD Fraction 1 (red), Fraction 2 (blue) and Fraction 3 (yellow) were analyzed with Venn Diagram. Numbers represent the amount of proteins belonging to the corresponding data sets.

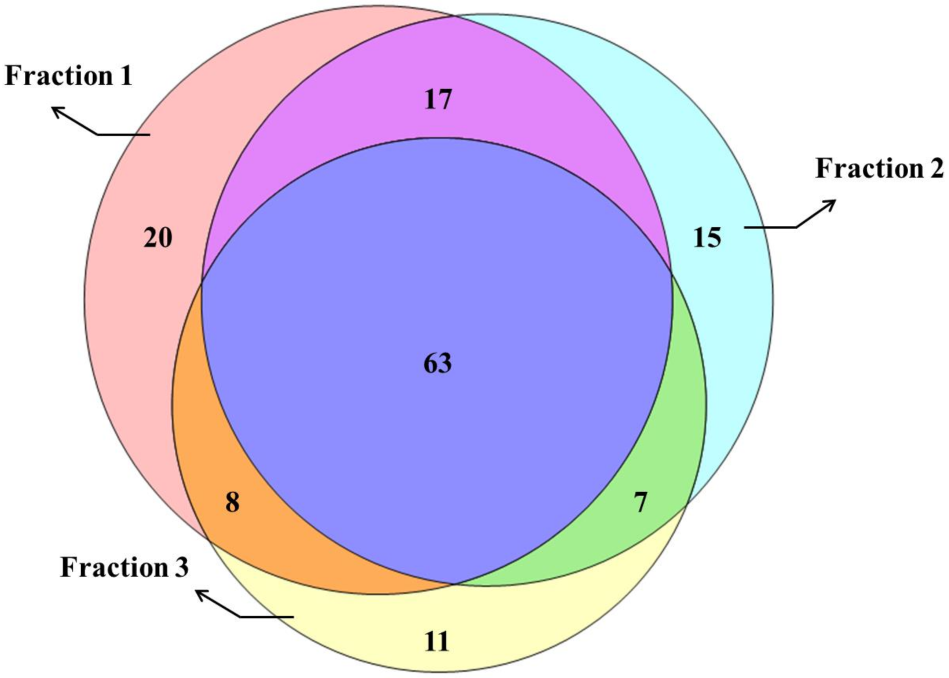

Supplemental Figure 3 Peptide numbers of proteins in LD subpopulations from CHO K2 cells.

The proteins identified were grouped according to their sub-cellular location or function as follows (A) lipid metabolism proteins, (B) ER proteins, (C) mitochondrial proteins and (D) Rab proteins. Peptide numbers of a specified protein from Fraction 1 (blue), Fraction 2 (red) and Fraction 3 (green) were counted respectively.

NSDHL, sterol-4-alpha-carboxylate 3-dehydrogenase (decarboxylating); DHRS1, dehydrogenase/reductase SDR family member 1; LRRC59, leucine-rich repeat-containing protein 59; AUP1, ancient ubiquitous protein 1; ALG5, dolichyl-phosphate beta-glucosyltransferase; StARD7, stAR-related lipid transfer protein 7.

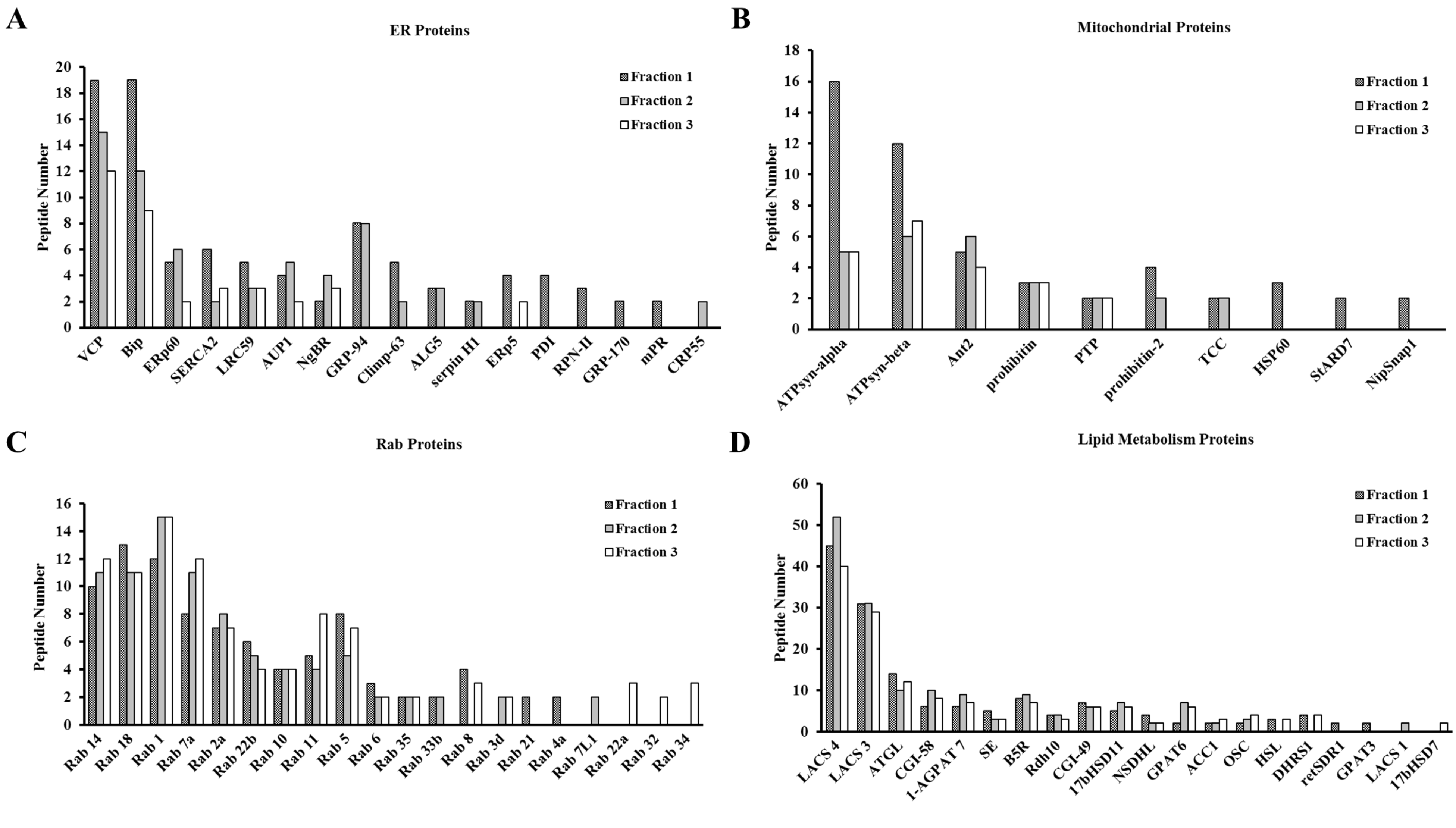
A
ER Proteins
Peptide Number
Fraction 1
Fraction 2
Fraction 3
VCP
Bip
ERp60
SERCA2
LRC59
AUP1
NgBR
GRP-94
Climp-63
ALG5
serpin H1
ERp5
PDI
RPN-II
GRP-170
mPR
CRP55
B
Mitochondrial Proteins
Peptide Number
Fraction 1
Fraction 2
Fraction 3
ATPsyn-alpha
ATPsyn-beta
Ant2
prohibitin
PTP
prohibitin-2
TCC
HSP60
StARD7
NipSnap1
C
Rab Proteins
Peptide Number
Fraction 1
Fraction 2
Fraction 3
Rab 14
Rab 18
Rab 1
Rab 7a
Rab 2a
Rab 22b
Rab 10
Rab 11
Rab 5
Rab 6
Rab 35
Rab 33b
Rab 8
Rab 3d
Rab 21
Rab 4a
Rab 7L1
Rab 22a
Rab 32
Rab 34
D
Lipid Metabolism Proteins
Peptide Number
Fraction 1
Fraction 2
Fraction 3
LACS 4
LACS 3
ATGL
CGI-58
1-AGPAT 7
SE
B5R
Rdh10
CGI-49
17bHSD11
NSDHL
GPAT6
ACC1
OSC
HSL
DHRS1
retSDR1
GPAT3
LACS 1
17bHSD7

Supplemental Reference

1 Zhang, H. *et al.* Proteome of skeletal muscle lipid droplet reveals association with mitochondria and apolipoprotein a-I. *J. Proteome Res.* **10**, 4757-4768 (2011).
